# Effect of Extended-Release Niacin/Laropiprant Combination on Plasma Adiponectin and Insulin Resistance in Chinese Patients with Dyslipidaemia

**DOI:** 10.1155/2015/154014

**Published:** 2015-04-29

**Authors:** Miao Hu, Ya-Ling Yang, Daisaku Masuda, Shizuya Yamashita, Brian Tomlinson

**Affiliations:** ^1^Department of Medicine & Therapeutics, The Chinese University of Hong Kong, Shatin, Hong Kong; ^2^Diabetes Center, Second Xiangya Hospital, Institute of Metabolism and Endocrinology, Key Laboratory of Diabetes Immunology, Ministry of Education, Central South University, Changsha, Hunan 410013, China; ^3^Department of Cardiovascular Medicine, Osaka University Graduate School of Medicine, Osaka 565-0871, Japan

## Abstract

*Objectives*. This study examined whether the increase of adiponectin associated with extended-release (ER) niacin/laropiprant combination attenuates the adverse effect of niacin on glucose and insulin resistance in Hong Kong Chinese patients with dyslipidaemia. *Methods*. Patients (*N* = 121) were treated with ER niacin/laropiprant 1 g/20 mg for 4 weeks and then the dose was doubled for an additional 8 weeks. Measurements of fasting lipids, glucose, insulin, and adiponectin were performed at baseline and during the study. *Results*. There were significant (*P* < 0.001) increases in glucose (9.4 ± 13.1%), insulin (70.2 ± 91.0%), HOMA-IR (87.8 ± 103.9%), and adiponectin (169.3 ± 111.6%). The increase in adiponectin was significantly associated with increase in glucose (*r* = 0.221, *P* < 0.05), insulin (*r* = 0.184, *P* < 0.05), and HOMA-IR (*r* = 0.237, *P* < 0.01) and the association remained significant after adjustment for changes in body weight or body fat mass. *Conclusion*. Treatment with ER niacin/laropiprant led to a significant increase in adiponectin levels but worsening of glucose levels and insulin resistance, and the increase in adiponectin and insulin resistance were correlated suggesting the increase in adiponectin did not ameliorate the deterioration in insulin resistance. Clinical trial is registered with number on WHO-ICTRP: ChiCTR-ONC-10001038.

## 1. Introduction

Adiponectin is an adipocytokine with anti-inflammatory, antiatherogenic, and insulin-sensitizing properties, which is produced predominantly in white adipose tissue [1–3]. It modulates a number of metabolic processes, including glucose regulation and fatty acid oxidation. Some clinical studies have shown that low adiponectin concentrations are associated with obesity, insulin resistance, metabolic syndrome, type 2 diabetes, and coronary artery disease and high plasma adiponectin levels are independently associated with a reduced risk of atherosclerotic cardiovascular disease (CVD) [4–6], whereas others reported opposite results or lack of associations [7, 8]. Meta-analyses of the clinical studies on the association of adiponectin level and CVD also showed controversial results [9–11]. Although there is evidence that elevated adiponectin levels are found in heart failure and some chronic inflammatory autoimmune diseases, such as systemic lupus erythematosus and rheumatoid arthritis, it is unclear whether the elevated adiponectin levels are involved in the pathogenesis or increased as a compensatory response to those conditions [2, 12].

Numerous* in vitro* and* in vivo* studies have demonstrated a direct beneficial action of adiponectin in metabolic and vascular disorders [1–3]. It has been proposed that adiponectin may represent a target molecule for manipulation of atherosclerotic diseases. Various therapeutic approaches targeted at increasing adiponectin levels, or its activity, are being explored and some of these approaches have achieved therapeutic benefits in animal models of metabolic diseases [13]. Weight loss and some cardiovascular pharmacotherapies increase adiponectin [3]. Several lipid-modifying drugs including statins, fibrates, niacin, and omega-3 fatty acids increase adiponectin levels with the greatest effects being produced by niacin [14].

Niacin is an old lipid-modifying drug which has favourable effects on all traditionally measured lipid parameters [15]. It showed benefits in preventing CVD and mortality in the 1970s when statins were not available [16]. However, two recent large outcome studies have shown that the extended-release (ER) niacin or the combination of extended-release (ER) niacin and laropiprant (a prostaglandin D2 receptor antagonist developed to reduce niacin-induced flushing) did not significantly further reduce the risk of the cardiovascular event endpoint in patients receiving intensive statin treatment [17, 18]. In addition to lipid-regulating actions, niacin has a broad range of effects: it increases serum concentrations of glucose, insulin, and uric acid and long-term treatment with niacin is associated with increased plasma free fatty acid (FFA) levels and some of these effects may offset the beneficial effects of niacin on cardiovascular risk [19]. Although the exact mechanisms for these wanted and unwanted effects of niacin are still not fully elucidated, it appears that some of them may be mediated directly via the niacin receptor, hydroxycarboxylic acid receptor 2 (HCAR2), previously known as G protein-coupled receptor (GPR) 109A [20–22].

HCAR2 is highly expressed in adipocytes, as well as other cell types including neutrophils, macrophages, keratinocytes, and Langerhans cells [23].* In vitro* and animal studies have shown that niacin stimulates adiponectin secretion in adipocytes through its receptor HCAR2 [24, 25]. It has been found that niacin promotes peroxisome proliferator-activated receptor gamma (PPAR*γ*) expression and transcriptional activation in macrophages via induced synthesis of prostaglandin associated with activation of the niacin receptor HCAR2 [26]. Zhao et al. also demonstrated that niacin dose-dependently enhanced PPAR*γ* and LXR*α* in rabbit adipocytes [27]. PPAR*γ* is a transcription factor that plays a central role in adipocyte biology and it regulates adiponectin gene expression, processing, and secretion [28], and thus it is conceivable that niacin may stimulate adiponectin secretion in adipocytes through the HCAR2-PPAR*γ* pathway.

In contrast to the thiazolidinediones, antidiabetic drugs that act by binding to and activating PPAR-*γ*, which improve insulin sensitivity by mechanisms which may involve increase in adiponectin [29], treatment with niacin often causes increased glucose levels and insulin resistance. The present study examined whether the increase of adiponectin associated with the niacin/laropiprant combination attenuates the adverse effect of niacin on glucose and insulin resistance.

## 2. Materials and Methods

### 2.1. Study Design and Patients

Patients included in this analysis were Hong Kong Han Chinese patients with dyslipidaemia, who had been recruited from the lipid clinic to participate in a pharmacogenetic study to examine the genetic determinants of the lipid responses to ER niacin/laropiprant combination. In brief, these patients were Hong Kong Chinese adults aged 18–85 years with primary hypercholesterolaemia or mixed dyslipidaemia with or without lipid-lowering therapy other than niacin. Patients had to be naïve to all lipid-lowering therapy or taking a stable dose of lipid-modifying therapy for at least four weeks for statins and ezetimibe or six weeks for fibrates. Patients were not eligible if they had a history of hypersensitivity to niacin or laropiprant or had significant hepatic or renal dysfunction, active peptic ulcer disease, or any contraindications to the ER niacin/laropiprant therapy. In this pharmacogenetic study, a total of 130 patients completed the study over a period of 12 weeks and during the study they received four weeks of ER niacin/laropiprant 1000/20 mg and then ER niacin/laropiprant 2000/40 mg for an additional eight weeks. Fasting blood samples for the laboratory analyses were collected at baseline and after the 12-week treatment period for the measurement of lipids, glucose, insulin, and adiponectin. Anthropometric measurements, including body weight, body height, waist circumference, hip circumference, and estimation of percentage body fat using an impedance device (TANITA Body Composition Analyzer BF-350, Japan), were performed at each study visit. The present analysis was performed in 121 patients with data on insulin, adiponectin, and FFA levels available. The study protocol was approved by the local Clinical Research Ethics Committee and all patients provided written informed consent.

### 2.2. Laboratory Analyses

The lipid, glucose, glycated haemoglobin (HbA1c), and laboratory safety parameters were measured by routine methods. Fasting FFA levels were determined using the enzymatic method. Homeostasis Model of Assessment-Insulin Resistance (HOMA-IR) was calculated using the following formula: HOMA-IR = fasting glucose (mmol/L) × fasting insulin (mU/L)/22.5 [30]. The adiponectin level was measured with the Human Adiponectin Immunoassay kit (catalogue number: 31010, Antibody and Immunoassay Services, The University of Hong Kong, Hong Kong). The detection limit is 1.56 ng/mL. The CVs (coefficients of variation) of intra- and interday assays were 3.78 and 4.80%, respectively. The insulin concentrations were measured by Insulin ELISA kit from DAKO (code K6219, Dako Denmark, Glostrup, Denmark). The absorbance was read in an ELISA plate reader (Micro-Quant, Bio-Tek Instruments, Inc., USA). The detection limit is 0.5 *μ*IU/mL. The CVs of intra- and interday assays were 6.7 and 7.5%, respectively.

### 2.3. Statistical Analysis

Data are shown as mean ± SD unless stated otherwise. Skewed data were logarithmically transformed before analysis. Changes in parameters examined during the study were evaluated by paired *t*-test. Associations between baseline lipids, glucose, insulin, and adiponectin and changes in these parameters were evaluated by Pearson's correlation test. Changes in lipids, glucose, insulin, and adiponectin in subjects with weight loss and weight gain were compared by Student's *t*-test or Mann-Whitney *U* test where appropriate. Backwards stepwise regression analysis was performed to examine the determinants of the percentage change in adiponectin in response to niacin/laropiprant. Differences were considered to be statistically significant, if the 2-sided *P* value was <0.05. Data were analyzed using SPSS version 17.0 (SPSS, Inc., Chicago, IL, USA).

## 3. Results

In 121 patients who completed the study with adiponectin levels available, 74 were males, 48 had type 2 diabetes, 92 had hypertension, and 75 were receiving other lipid-lowering treatments. Male subjects had a significantly lower baseline adiponectin level than females (9.1 ± 5.9 *μ*g/mL versus 12.3 ± 7.2 *μ*g/mL, *P* < 0.01). At baseline, the adiponectin levels were inversely correlated with the body weight (*r* = −0.281, *P* < 0.01), body fat mass (*r* = −0.213, *P* < 0.05), and waist circumference (*r* = −0.277, *P* < 0.01). Baseline fasting glucose (*r* = −0.199, *P* < 0.05), HbA1c (*r* = −0.208, *P* < 0.05), insulin (*r* = −0.36, *P* < 0.001), and HOMA-IR (*r* = −0.384, *P* < 0.001) were inversely correlated with adiponectin concentrations, whereas the HDL-C levels were significantly positively associated with the adiponectin levels (*r* = 0.439, *P* < 0.001).

**Figure 1:**
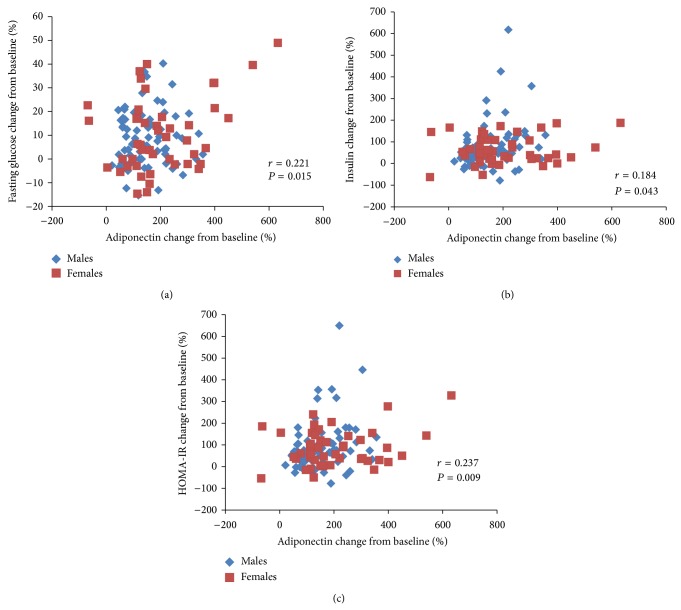
Associations between changes in adiponectin and changes in fasting glucose (a), insulin (b), and the Homeostasis Model of Assessment-Insulin Resistance (HOMA-IR) (c) with ER niacin/laropiprant.

After treatment with ER niacin/laropiprant for 12 weeks, there were significant (*P* < 0.001) increases in HDL-C (23.3 ± 22.7%), glucose (9.4 ± 13.1%), insulin (70.2 ± 91.0%), HOMA-IR (87.8 ± 103.9%), FFA (74.5 ± 105.3%), and adiponectin (169.3 ± 111.6%) and decreases in triglycerides (−31.8 ± 22.7%) and LDL-C (−19.8 ± 26.2%) levels (Table 1). These changes remained significant after adjustment for the changes in body weight (−0.7 ± 2.0%) or fat mass (−5.4 ± 8.7%) during the study. Adiponectin increased in 119 out of the 121 patients with niacin therapy. In univariate analysis, there was a significant correlation between the increase in adiponectin and increase in glucose, insulin, or HOMO-IR (Figure 1) and the association remained significant after adjustment for changes in body weight or body fat mass. There was no statistically significant difference in adiponectin changes between subjects with weight gain and weight loss (Table 2) or between subjects with body fat loss (*n* = 83) versus those with body fat gain (*n* = 37) (180.5 ± 121.9% versus 142.6 ± 79.4%, *P* = 0.083), but subjects with weight loss had less increases in glucose, insulin, and HOMO-IR than those with weight gain (Table 2). Multivariate regression analysis revealed that female gender, lower baseline body fat mass, not having hypertension, greater increases in glucose, and greater reductions in triglycerides and body fat mass during the study were associated with greater increases in adiponectin levels (Table 3) and these factors explained 31.3% of variance in the adiponectin change in response to niacin/laropiprant.

Stepwise multiple linear regression analysis showed that percentage changes in adiponectin (*P* < 0.01), baseline HbA1C level (*P* < 0.01), baseline glucose levels (*P* < 0.05), and changes in body weight (*P* < 0.05) were associated with changes in fasting glucose levels and these factors accounted for 13.5% of the variance in the changes in glucose levels in response to niacin/laropiprant (data not shown).

## 4. Discussion

Niacin has a wide range of effects including increasing circulating levels of adiponectin, probably via stimulating adiponectin secretion by binding to the HCAR2 receptor in adipose tissue [24, 25] and this may contribute to its anti-inflammatory effect on atherosclerosis [25]. It has been demonstrated that adiponectin improves insulin sensitivity through inhibition of hepatic glucose production and enhancing glucose uptake in muscle, and increasing fatty acid oxidation in liver and skeletal muscle by activation of AMPK and PPAR*α* mediated by the adiponectin receptors 1 and 2 [31, 32].

The present study examined whether the niacin-induced increase in adiponectin levels had any impact on the effect of niacin on plasma glucose levels and insulin resistance. We found that the insulin levels and HOMA-IR were inversely associated with adiponectin levels at baseline and after treatment with niacin/laropiprant in the univariate analysis and after adjustment for baseline body weight or body fat mass. However, counterintuitively the increases in adiponectin levels with niacin/laropiprant were associated with increases in fasting glucose, insulin, and HOMA-IR and the association between the changes in adiponectin and changes in glucose remained significant after adjustment for the other confounding factors. Furthermore, greater increases in adiponectin were associated with lower baseline body fat mass and greater reduction in triglycerides in response to niacin/laropiprant. As the degree of reduction in triglycerides and increase in adiponectin and glucose with niacin are dose-dependent, the inverse correlation between baseline body fat mass and changes in these parameters may be related to a common underlying pharmacokinetic effect as plasma and tissue levels of niacin are likely to be higher in patients with lower body weight.

Fraterrigo et al. reported similar observations in 9 obese subjects with nonalcoholic fatty liver disease in a small study using a hyperinsulinemic-euglycemic clamp procedure to assess muscle insulin sensitivity before and after 16 weeks of niacin therapy [33]. They also found that the deterioration in glucose disposal was inversely correlated with the niacin-induced increase in plasma adiponectin concentration (*r* = 0.67, *P* = 0.05). The paradoxical effect of niacin/laropiprant on adiponectin and glucose levels/glucose disposal observed in our study and in Fraterrigo's study indicates that either the increased adiponectin levels with niacin treatment do not produce a beneficial insulin-sensitizing effect, as usually observed in physiological conditions, or an adiponectin-independent effect produced by niacin decreased insulin sensitivity to a greater magnitude than the beneficial effect of adiponectin upon insulin sensitivity.

The interactions between insulin and adiponectin are complex. Animal studies suggested that insulin negatively regulates the expression levels of adiponectin receptors and adiponectin sensitivity via the phosphoinositide 3-kinase (PI3-kinase)/forkhead box O1- (Foxo1-) dependent pathway [34]. Insulin resistance can result in hyperadiponectinaemia and adiponectin resistance in insulin receptor transgenic/knockout mice [35], in adipocyte-specific insulin receptor (Insr) knockout mice [36] or in mice with muscle-specific insulin resistance due to transdominant inhibition of Insr and IGF-1 receptors [37], suggesting that adiponectin production is controlled in response to changes in systemic insulin sensitivity. These data raise the possibility that the increased insulin levels/insulin resistance induced by niacin may downregulate adiponectin signaling and attenuate the insulin-sensitizing actions of adiponectin.

The exact mechanism responsible for the deleterious effect of niacin therapy on insulin sensitivity is still unclear, but it may be related to the FFA rebound and increased basal plasma FFA levels with long-term niacin therapy. It has been proposed that niacin-induced activation of the transcription factor FOXO1 in insulin-sensitive tissues, including the liver, skeletal muscle, heart, and adipose tissue, may be responsible for the development of insulin resistance during niacin therapy as some FOXO1 target genes (e.g., pyruvate dehydrogenase kinase Isoform 4, glucose 6-phosphatase, phosphoenolpyruvate carboxykinase, PPAR*γ* coactivator 1, etc.) are known to regulate blood-glucose control and/or insulin sensitivity [19, 38].

Greater increase in adiponectin was observed in less obese individuals receiving niacin therapy in previous studies [39] and this was also found in the present study. In this open label study, there was a small but significant reduction in body weight and body fat mass. It is known that reduction in adipose tissue mass increases adiponectin levels. Indeed, a greater reduction in body fat mass was associated with a greater increase in adiponectin during the study suggesting an additive effect of fat loss and niacin therapy on increasing adiponectin concentrations. Although the changes in body weight/fat mass in this study may confound the results for the association between changes in adiponectin and changes in glucose with niacin/laropiprant therapy, the association remains significant after adjustment for changes in body weight or body fat mass and a similar association was also observed in obese subjects without weight changes after niacin therapy [33].

Clinical and experimental studies demonstrate the relationship of lower plasma adiponectin concentrations with hypertension [40]. It appears that adiponectin regulates blood pressure by stimulating the production of NO and vasodilatation and inhibiting the activation of the sympathetic nervous system through central actions [41]. In the present study, there was no significant difference in the baseline adiponectin concentrations in patients with and without hypertension, and this may be related to the antihypertensive treatments received in patients with hypertension as some antihypertensive drugs, for example, angiotensin II type 1 receptor blockers (ARBs), are known to increase plasma adiponectin concentrations, probably through PPAR-*γ* activation [40, 42]. Interestingly, this study showed that patients with hypertension had less increase in adiponectin levels after treatment with niacin/laropiprant than those without hypertension, suggesting a complex interaction between hypertension, antihypertensive treatments, niacin, and adiponectin concentrations, and this warrants further evaluation.

Although this is the first report of the effect of ER niacin/laropiprant on adiponectin and insulin resistance levels in a Chinese population, this study has several limitations to be considered. Firstly, adiponectin exists in the circulation as three isoforms: trimers, hexamers, and high molecular weight (HMW) forms, potentially with different biological activity [43, 44], but the present study only measured the total adiponectin concentration. However, the HMW isoform appears to be the most biologically active form which is most affected by treatment with niacin (88% increase versus 35% and 33% increase for the low- and medium-molecular weight adiponectin, resp.) and the niacin-associated deterioration of insulin sensitivity, as assessed by the HOMA-IR, occurs even in subgroups with the greatest increase of HMW adiponectin [39]. In the present study, circulating total adiponectin levels were measured with a sandwich ELISA kit established locally. A previous cohort study using this test kit showed the typical inverse correlations between plasma total adiponectin level and fasting glucose, insulin, and HOMA-IR in Hong Kong Chinese subjects [45]. Overall, this limitation probably would not affect the result of the study. Furthermore, the uncontrolled study design and change in body fat mass during the study make it difficult to assess the exact effect of niacin/laropiprant on adiponectin, glucose, and insulin levels. In addition, the association between changes in glucose and adiponectin in this study was observed with the combination of ER niacin and laropiprant and it is unknown whether laropiprant has any effect on adiponectin. In phase III studies, ER niacin/laropiprant and ER niacin alone had similar effects on increasing plasma glucose levels [46] and the previous small study reported a similar association of increases in adiponectin and insulin resistance with ER niacin taken alone [33], and thus laropiprant may be unlikely to affect the association between changes in plasma adiponectin levels and insulin resistance. The mechanism underlying the association between changes in glucose and adiponectin concentration observed in the study requires further investigation.

In conclusion, the present study demonstrated that treatment with ER niacin/laropiprant led to a significant increase in adiponectin levels but worsening of insulin resistance, which were positively associated. This result suggested that increased adiponectin levels induced by niacin either do not produce a beneficial insulin-sensitizing effect or do not overcome the deleterious effect of niacin therapy on insulin sensitivity through adiponectin-independent pathways. Future studies are needed to evaluate the underlying mechanisms and to examine whether this increase in adiponectin concentration with niacin treatment has any other benefits in terms of anti-inflammatory or antiatherogenic effects.

## Figures and Tables

**Table 1:** Changes in anthropometric and biochemical parameters during the study.

	Baseline	After treatment	% changes	*P*
Body weight (kg)	73.1 ± 15.5	72.5 ± 15.2	−0.7 ± 2.0	<0.001
Body fat (%)	31.2 ± 7.2	29.8 ± 7.4	−4.7 ± 8.4	<0.001
Fat mass (kg)	22.8 ± 7.5	21.6 ± 7.5	−5.4 ± 8.7	<0.001
Lean body mass (kg)	50.2 ± 11.4	50.8 ± 11.4	1.5 ± 4.3	0.02
BMI (kg/m^2^)	27.5 ± 4.4	27.3 ± 4.3	−0.7 ± 2.3	<0.001
Waist circumference (cm)	92.4 ± 11.1	92.3 ± 11.7	−0.2 ± 3.9	0.633
Total cholesterol (mmol/L)	5.39 ± 1.00	4.56 ± 0.96	−14.5 ± 14.8	<0.001
HDL-C (mmol/L)	1.19 ± 0.27	1.45 ± 0.38	23.3 ± 22.7	<0.001
Triglycerides (mmol/L)	2.29 ± 1.25	1.44 ± 0.84	−31.8 ± 29.2	<0.001
LDL-C (mmol/L)	3.19 ± 0.92	2.46 ± 0.83	−19.8 ± 26.2	<0.001
Non-HDL (mmol/L)	4.20 ± 0.95	3.12 ± 0.93	−24.7 ± 18.8	<0.001
Free fatty acids (*μ*Eq/L)	481.8 ± 204.3	745.3 ± 351.5	74.5 ± 105.3	<0.001
Fasting glucose (mmol/L)	5.62 ± 1.12	6.15 ± 1.47	9.4 ± 13.1	<0.001
HbA1C (%)	6.33 ± 0.72	6.63 ± 0.87	4.8 ± 7.4	<0.001
Fasting insulin, mU/L	11.95 ± 8.54	17.34 ± 10.44	70.2 ± 91.0	<0.001
HOMA-IR	3.03 ± 2.31	4.78 ± 3.07	87.8 ± 103.9	<0.001
Adiponectin, *µ*g/mL	10.32 ± 6.60	28.11 ± 22.4	169.3 ± 111.6	<0.001

BMI: body mass index; HbA1C: glycated haemoglobin; HDL-C: high-density lipoprotein cholesterol; HOMA-IR: Homeostasis Model of Assessment-Insulin Resistance; LDL-C: low-density lipoprotein cholesterol.

**Table 2:** Changes in body weight and blood biochemical parameters in patients with weight gain and weight loss.

Parameters	Weight gain (*n* = 42)	Weight loss (*n* = 79)	*P*
Weight changes (kg)	1.0 ± 0.8	−1.4 ± 1.0	<0.001
% changes in body weight	1.4 ± 1.2	−1.8 ± 1.3	<0.001
% changes in adiponectin	165.6 ± 133.7	171.2 ± 98.7	0.327
% changes in fasting glucose	14.6 ± 13.3	6.7 ± 12.2	0.001
% changes in HbA1c	5.2 ± 5.4	4.7 ± 8.3	0.205
% changes in insulin	80.7 ± 58.1	64.6 ± 104.3	0.007
% changes in HOMA-IR	109.3 ± 82.3	76.3 ± 112.5	0.001
% changes in FFA	150.9 ± 72.0	87.1 ± 117.8	0.201
% changes in total cholesterol	−16.0 ± 14.6	−13.6 ± 14.9	0.403
% changes in HDL-C	20.4 ± 23.4	24.9 ± 22.3	0.336
% changes in triglycerides	−31.1 ± 36.6	−32.2 ± 24.6	0.533
% changes in LDL-C	−21.0 ± 24.1	−19.2 ± 27.4	0.909

FFA: free fatty acids; HbA1c: glycated haemoglobin; HDL-C: high-density lipoprotein cholesterol; HOMA-IR: Homeostasis Model of Assessment-Insulin Resistance; LDL-C: low-density lipoprotein cholesterol.

**Table 3:** Predictors of the percentage change in adiponectin in response to ER niacin/laropiprant.

Variables	% change in adiponectin			
	Univariate analysis		Multivariate analysis	
	*r*	*P*	*r*	*P*
Age, years	0.197	0.03	0.088	0.353
Gender (1 = male, 2 = female)	0.228	0.012	0.215	0.006
Baseline body weight, kg	−0.387	<0.001	0.068	0.475
Baseline body fat, %	−0.135	0.139		
Baseline body fat mass, kg	−0.351	<0.001	−0.265	0.001
Baseline BMI, kg/m^2^	−0.389	<0.001	−0.003	0.972
Baseline waist circumference, cm	−0.421	<0.001	−0.04	0.672
Hypertension (1 = Yes, 2 = No)	0.273	0.002	0.207	0.008
Diabetes (1 = Yes, 2 = No)	−0.007	0.938		
Baseline adiponectin levels, *µ*g/mL	0.043	0.637		
Baseline glucose levels, mmol/L	−0.063	0.493		
Baseline insulin levels, mU/L	−0.272	0.003	−0.073	0.44
Baseline HOMA-IR	−0.276	0.002	0.061	0.516
% change in body weight	−0.078	0.396		
% change in body fat mass	−0.235	0.01	−0.238	0.003
% change in lean body mass	0.14	0.126		
% change in fasting glucose	0.221	0.015	0.189	0.015
% change in HbA1c	0.045	0.624		
% change in insulin	0.184	0.043	∗	
% change in HOMA-IR	0.237	0.009	0.037	0.697
% change in FFA	0.083	0.364		
% change in HDL-C	0.121	0.186		
% change in triglycerides	−0.303	<0.001	−0.176	0.027
% change in LDL-C	−0.256	0.005	−0.105	0.268

^∗^Not included due to collinearity with % change in HOMA-IR.

BMI: body mass index; FFA: free fatty acids; HbA1C: glycated haemoglobin; HDL-C: high-density lipoprotein cholesterol; HOMA-IR: Homeostasis Model of Assessment-Insulin Resistance; LDL-C: low-density lipoprotein cholesterol.
